# A Novel ATP1A2 Gene Variant Associated With Pure Sporadic Hemiplegic Migraine Improved After Patent Foramen Ovale Closure: A Case Report

**DOI:** 10.3389/fneur.2018.00332

**Published:** 2018-05-17

**Authors:** Armando Perrotta, Stefano Gambardella, Anna Ambrosini, Maria Grazia Anastasio, Veronica Albano, Francesco Fornai, Francesco Pierelli

**Affiliations:** ^1^IRCCS Neuromed, Pozzilli, Italy; ^2^Department of Translational Research and New Technologies in Medicine and Surgery, University of Pisa, Pisa, Italy; ^3^Unit of Neurorehabilitation, Department of Medical-Surgical Sciences and Biotechnologies, Sapienza University of Rome, Istituto Chirurgico Ortopedico Traumatologico (ICOT), Latina, Italy

**Keywords:** sporadic hemiplegic migraine, migraine aura, atrial septal aneurysm, incomplete penetrance, vascular factors

## Abstract

We describe the case of one patient with pure sporadic hemiplegic migraine (SHM) with a novel ATP1A2 gene variant and a large patent foramen ovale (PFO) with atrial septal aneurysm. In hemiplegic migraine (HM), the relationship between incomplete penetrance, environmental triggers, and phenotypic expression is underdetermined. A genetic evaluation of the proband was requested for the HM associated genes and extended to the members of his family. Genetic analysis revealed a never described before ATP1A2 gene mutation, inherited by his father, who never experienced motor aura but only typical visual aura. The proband—but not his father—was also affected by a large PFO with atrial septal aneurysm. SHM patient showed a marked reduction in motor aura episodes per year in the 12 months following the PFO percutaneous closure, followed by a complete remission from attacks at least in the following 24 months. We speculated that as well as incomplete penetrance of the novel mutation and natural history of the disease, an additional pathological condition such as the PFO could contribute to the phenotypical expression in this case of HM.

## Introduction

According to the International Classification of Headache Disorders 3 beta version (1), hemiplegic migraine (HM) is a rare form of migraine with aura, characterized by visual, sensory, and/or brainstem aura symptoms accompanied by transient unilateral motor weakness of variable severity and duration, followed by migraine headache. HM represents a clinically heterogeneous disorder, which varies from “pure” to complex forms, which may associate with permanent ataxia, infantile convulsions, epileptic seizures, cerebral edema, and coma after minor head trauma and mental retardation (2). In addition, most of the patients also complaint of attacks of typical aura with or without migraine headache, or less, migraine without aura, not-associated with motor aura (3).

Hemiplegic migraine has two recognized forms: (i) familial HM (FHM), when at least one first- or second-degree patients’ relative is affected; and (ii) sporadic hemiplegic migraine (SHM), which is undistinguishable from familial forms clinically, but no relative fulfils criteria for FHM (1). SHM can be caused by a novel mutation in a gene that causes the familial form or by inheritance of a gene mutation from a parent with “non hemiplegic” typical aura with migraine headache or migraine without aura (4). So far, mutations in three different genes, CACNA1A, ATP1A2, and SCN1A, have been described to cause FHM and they are referred as FHM1, FHM2, and FHM3, respectively (2). Mutations in CACNA1A and ATP1A2 have been reported in some SHM subjects, while SCN1A mutations have never been reported so far in this condition (4, 5). Since all these genes encodes for sub-units of neuronal and glial membrane ion channels, both FHM and SHM are considered cerebral channelopathies, in which the effects of high concentrations of glutamate and/or extracellular K^+^ interfere with cortical excitability.

In SHM, the relationship between incomplete penetrance, external and internal environmental triggers, and phenotypic expression is underdetermined. Similarly, the role of vascular factors in the cascade of events involved in the pathogenesis of non-hemiplegic forms of migraine aura (MA) has been largely explored, however, without concordant results. More recently, in a large series of patients, an association between MA and patent foramen ovale (PFO) has been identified in presence of a concomitant atrial septal aneurysm (6), animating the discussion about the role of the vascular factors in the pathogenesis and/or phenotypic expression of MA.

Here, we describe the case of a 23-year-old student with a history of infantile convulsions and HM appearing as sporadic, who carries a novel mutation in the ATP1A2 gene. The mutation was inherited from his father, who never suffered from HM attacks, but only from a typical non-hemiplegic visual MA with migraine headache. The proband was also affected by the presence of a large PFO with atrial septal aneurysm and experienced a significant reduction of HM attacks after PFO closure.

## Case Report

The patient came to consultation complaining of recurrent episodes of motor and visual disturbances, which occurred in association with headache. The first episode occurred at the age of 9 years and consisted of visual aura and migraine headache, followed by paresis of the right limbs, which lasted a few hours. In the following years, similar episodes with a frequency of 10 or 11 per year occurred with paresis affecting either the left or the right side. According to the patient’s description, he had episodes of scintillating scotomas in the right/left visual hemifield, which lasted about 20 min, and were followed by typical pulsating fronto-temporal left/right headache, which was exacerbated by movements, lasting about 24–48 h with photophobia, phonophobia, nausea, and vomiting. Paresis and paresthesia started in the right/left hand and progressively extended to the homolateral side of the face and leg, accompanied by no better specified speech disturbances. Motor symptoms reached the maximum severity in 30 min, lasted a few hours, and resolved spontaneously. Before he came to our observation, he was diagnosed with visual and somatosensory aura with migraine headache (and/or probable HM) and no preventive but only symptomatic treatments were advised. The proband did not suffer from any other form of migraine or primary headache.

According to the increased incidence of PFO in subject with MA (6), when he was 18 years old, was screened and diagnosed with large PFO and atrial septal aneurysm and at the age of 19 years, he underwent a successful percutaneous closure surgery. Interestingly, motor and visual aura episodes frequency was reduced from several to two episodes per year in the next 12 months after surgical closure of the PFO, followed by a complete remission in the next 2 years, when he came to our observation. He took acetyl-salicylic acid 100 mg per day only during the 6 months following the PFO closure, and he does not currently assume any treatment and does not complain of any type of headache. Except from the PFO closure, there was no noticeable change in his past clinical history.

The neurological exam, electroencephalogram, and brain MRI were normal. A full hematologic profile, including coagulation screening, revealed negative results. In light of the clinical features, the patient was diagnosed as suffering from SHM. A genetic evaluation of the proband was requested for the HM associated genes, CACNA1A, ATP1A2, and SCN1A. The genetic and clinical evaluation was extended to the proband’s parents (the proband is a single child) and to other members of his family, after a written informed consent was obtained from the participants. A written informed consent was obtained from the proband for the publication of this case.

## Genetic Analysis

DNA was extracted from peripheral blood using standard techniques. A gene panel targeting ATP1A2, CACNA1A, and SCN1a genes was designed using Illumina Design Studio (San Diego, CA, USA). Sequencing of the target regions (coding regions and splice sites) was performed with MiSeq platform (Illumina). Annotation and characterization of variants was performed with Variant Studio. The manual examination and visualization of the sequence data was performed by the Integrative Genomics Viewer v.2.3. Mutations were re-sequenced by Sanger sequencing (ABI 3130xl Genetic Analyzer, Applied Biosystems).

## Results

Sequence analysis detected the variant, which consists of a heterozygous G-to-T transition at the nucleotide position 2810 of the coding sequence of the gene ATP1A2 (transcription variant NM_000702.3). This single base change in the exon 20 results in the substitution of arginine with leucine 937 (Arg937Leu). The presence of this variant was evaluated in his parents, and it was detected in his father, who never had HM attacks, but suffered from a typical non-hemiplegic visual aura with migraine headache. The father underwent to echocardiography, which excluded the presence of a right-to-left shunt. As declared by proband’s parents, no one else in the proband’s family was suffering from any other form of headache. However, no one, except his parents, was available for clinical interview or genetical analysis.

## Discussion

To date, more than 80 ATP1A2 mutations have been reported in FHM2, SHM, and migraine with brainstem aura patients (2, 4). Here a novel mutation, p.Arg937Leu (c.2810g > t) into the M8–M9 coding domain of ATP1A2 is reported in a subject affected by SHM. Interestingly, Riant et al. (7) described in a family with FHM a mutation affecting the same amino acid, causing an Arginine to Proline substitution, p.Arg937Pro (c.2810G > A). This concordance supports a pathogenetic role of the novel mutation that we found in our proband, rather than a rare not pathological polymorphism. Unfortunately, no clinical data are available for a comparison between the family described by Riant et al. (7) with the subjects described here.

However, as this novel mutation was inherited from the father, who has MA but not HM, its causal role in HM cannot be established with certainty. This variant could be a causative mutation with incomplete penetrance. Otherwise, this variant could produce, in various contexts, different clinical phenotypes. In fact, most of ATP1A2 *de novo* mutations are reported in patients with SHM with additional neurological findings and early onset (4, 5) and mutations in this gene have been associated with different clinical phenotypes, including non-hemiplegic auras (8). In this scenario, only a few reports describe the clinical spectrum of carriers of the same mutation, and this hypothesis requires several genotype–phenotype correlations in HM patients.

In our case, although we cannot exclude a further mutation in unidentified genes, as reported for most SHM (9), the phenotype of this patient could be due to genetic factors and concurrent additional pathological conditions such as the presence of a PFO with substantial hemodynamic effects.

Remarkably, in this patient, the surgical closure of PFO was associated with sudden improvement of the frequency of HM attacks, dropped off from several per years to stable remission in 12 months, which cannot be due to the effect of the ASA treatment, stopped after 6 months. Data about a causal relationship between PFO and HM are lacking, but previous cases of sporadic form of HM in which a PFO was found and which improved after the surgical closure, are reported (10, 11).

Current literature is largely discordant about a linkage between PFO and MA, though an association between MA and PFO has been recently identified in presence of a concomitant atrial septal aneurysm (6), as in our case. Anyhow, the role of vascular factors in the cascade of events involved in the aura pathogenesis is only speculative to date.

Therefore, despite the improvement of neurological symptoms following PFO closure is already described in some reports, this remains to be documented consistently (12).

On these bases, we can only speculate that the novel mutation identified here joined with a specific pathological condition, represented by a significant right-to-left shunt, could have contributed to produce the clinical aura expression or its recurrence. The hypothesis that PFO may play a role as co-factor may include the chance that this novel mutation may possess incomplete penetrance as supported by the absence of hemiplegic aura symptoms in the proband’s father, who was not affected by PFO.

We are aware that the natural history of HM does not allow to reach such a strong improvement as the one we observed in the case object of the study, which further support the concurrency of PFO and of the ATP1A2 gene described mutation in causing the symptoms or their recurrence. However, in the natural history of the HM, a progressive decrease in frequency and severity at adulthood could occur, as well as long interval without attacks are possible.

## Concluding Remarks

In conclusion, we identified in a subject with SHM a novel mutation, p.Arg937Leu (c.2810g > t) into the M8–M9 coding domain of ATP1A2 gene, affecting the same amino acid previously described in a family with FHM (7). In addition, as our subject was diagnosed with large PFO and atrial septal aneurysm and we observed a significant improvement in hemiplegic aura recurrence following the PFO closure, we hypothesized that PFO and related vascular factors could have played as concomitant pathogenic factors influencing the clinical aura expression and/or its recurrence. However, the role of PFO and hemodynamic factors in the phenotypic expression of the aura in both MA and HM needs to be addressed by specific studies on larger series.

## Ethics Statement

A written informed consent was obtained by the proband and his parent. No investigation or intervention was performed outside routine clinical care for this patient. As this is a case report, without experimental intervention into routine care, no formal research ethics approval is required.

## Author Contributions

AP and SG: conception, design, and drafting the manuscript. MA and VA: acquisition of data. AP, SG, AA, FF, and FP: analysis and interpretation of data, revision of the manuscript, and final approval.

## Conflict of Interest Statement

The authors declare that the research was conducted in the absence of any commercial or financial relationships that could be construed as a potential conflict of interest.

## References

[1] Headache Classification Committee of the International Headache Society (IHS). The international classification of headache disorders, 3rd edition (beta version). Cephalalgia (2013) 33:629–808. 10.1177/0333102413485658 23771276

[2] Russell MB Ducros A. Sporadic and familial hemiplegic migraine: pathophysiological mechanisms, clinical characteristics, diagnosis, and management. Lancet Neurol (2011) 10:457–70. 10.1016/S1474-4422(11)70048-5 21458376

[3] Thomsen LL Ostergaard E Olesen J Russell MB. Evidence for a separate type of migraine with aura: sporadic hemiplegic migraine. Neurology (2003) 60:595–601. 10.1212/01.WNL.0000046524.25369.7D 12601098

[4] de Vries B Freilinger T Vanmolkot KR Koenderink JB Stam AH Terwindt GM Systematic analysis of three FHM genes in 39 sporadic patients with hemiplegic migraine. Neurology (2007) 69:2170–6. 10.1212/01.wnl.0000295670.01629.5a 18056581

[5] Riant F Ducros A Ploton C Barbance C Depienne C Tournier-Lasserve E De novo mutations in ATP1A2 and CACNA1A are frequent in early onset sporadic hemiplegic migraine. Neurology (2010) 75:967–72. 10.1212/WNL.0b013e3181f25e8f 20837964

[6] Snijder RJ Luermans JG de Heij AH Thijs V Schonewille WJ Van De Bruaene A Patent foramen ovale with atrial septal aneurysm is strongly associated with migraine with aura: a large observational study. J Am Heart Assoc (2016) 5(12):e003771. 10.1161/JAHA.116.003771 27930349 PMC5210450

[7] Riant F De Fusco M Aridon P Ducros A Ploton C Marchelli F ATP1A2 mutations in 11 families with familial hemiplegic migraine. Hum Mutat (2005) 26:281. 10.1002/humu.9361 16088919

[8] Ambrosini A D’Onofrio M Grieco GS Di Mambro A Montagna G Fortini D Familial basilar migraine associated with a new mutation in the ATP1A2 gene. Neurology (2005) 65:1826–8. 10.1212/01.wnl.0000187072.71931.c0 16344534

[9] Thomsen L Oestergaard E Bjornsson A Stefansson H Fasquel AC Gulcher J Screen for CACNA1A and ATP1A2 mutations in sporadic hemiplegic migraine patients. Cephalalgia (2008) 28:914–21. 10.1111/j.1468-2982.2008.01599.x 18513263

[10] Brighina F Gurgone G Gaglio RM Palermo A Cosentino G Fierro B. A case of atypical sporadic hemiplegic migraine associated with PFO and hypoplasia of vertebro-basilar system. J Headache Pain (2009) 10:303–6. 10.1007/s10194-009-0125-3 19421707 PMC3451752

[11] Lemka M Pienczk-Reclawowicz K Pilarska E Szmuda M Cessation of sporadic hemiplegic migraine attacks after patent foramen ovale closure. Dev Med Child Neurol (2009) 51:923–4. 10.1111/j.1469-8749.2009.03466.x 19758362

[12] Mattle HP Evers S Hildick-Smith D Becker WJ Baumgartner H Chataway J Percutaneous closure of patent foramen ovale in migraine with aura, a randomized controlled trial. Eur Heart J (2016) 37:2029–36. 10.1093/eurheartj/ehw027 26908949

